# Stent-Assisted Coiling of Ruptured and Incidental Aneurysms of the Intracranial Circulation Using Moderately Flow-Redirecting, Braided Leo Stents—Initial Experience in 39 Patients

**DOI:** 10.3389/fneur.2017.00602

**Published:** 2017-11-14

**Authors:** Peter Voigt, Stefan Schob, Robert Jantschke, Ulf Nestler, Matthias Krause, David Weise, Donald Lobsien, Karl-Titus Hoffmann, Ulf Quäschling

**Affiliations:** ^1^Department of Neuroradiology, Leipzig University Hospital, Leipzig, Germany; ^2^Department of Neurosurgery, Leipzig University Hospital, Leipzig, Germany; ^3^Department of Neurology, Leipzig University Hospital, Leipzig, Germany

**Keywords:** endovascular therapy, aneurysm, coiling, stenting, flow diversion treatment

## Abstract

**Background:**

Flow diversion (FD)—a young technique using stents with highly increased surface coverage—was introduced to treat complex aneurysms without intra-aneurysmal material placement and has amended the spectrum of endovascular techniques such as stent-assisted coil occlusion considerably. However, ischemic complications, a common side effect in FD, occur more frequently compared with the conventional endovascular approaches and certainly limit the indication of this technique. Our study aimed to investigate the feasibility and efficacy of stent-assisted coiling using low profile self-expandable stents, which exhibit only moderate flow-redirecting properties and therefore represent a combination of hemodynamic endovascular and occlusive endosaccular therapy.

**Materials and methods:**

39 Patients were included in our retrospective study. Occlusion rates were assessed 6 months after the procedure in a total of 27 cases using the Raymond scale.

**Results:**

Complete occlusion (Raymond I) was achieved in 24/27 aneurysms. Small neck remnants (Raymond II) were evident in 3/27 aneurysms. There were no cases with sac remnant or complete persistence of aneurysmal filling (Raymond III and IV).

**Conclusion:**

Our study demonstrates interventional treatment of intracranial aneurysms using flow-redirecting stent-assisted coiling to be technically feasible and highly effective in aneurysmal occlusion. We believe that this approach is outstanding in the prevention of long-term aneurysmal reperfusion and exhibits a more acceptable risk profile than highly efficient FD techniques.

## Introduction

Although stenting and coiling (SAC) as well as balloon-assisted coiling (BAC) are both longstanding, well established endovascular approaches for the treatment of complex cases of intracranial aneurysms (1–3), recanalization of, respectively, treated aneurysms still occurs in a number of cases (4). Hence, a subsidiary endovascular approach, flow diversion (FD), was introduced to further enhance endovascular aneurysm therapy (5). FD has been established as a successful technique to neutralize wide-neck sidewall aneurysms with saccular configuration, selected bifurcation aneurysms, fusiform aneurysms, and dissecting aneurysms of the intracranial circulation (6–12). FD uses devices with significantly increased material coverage in comparison with conventional stents, aiming to reconstruct the parent vessel whilst excluding the aneurysm from the circulation (5, 13, 14). This new technique has already become the preferential treatment method of choice for a number of types of aneurysms (15). However, in the context of FD, sufficient dual platelet inhibition is an essential prerequisite to prevent ischemic complications, especially during the period of neointima formation shortly after flow diverter implantation (9, 16). Determined by the level of porosity of the device, blood flow is significantly altered not only within the aneurysm sac but also in the parent vessel, which results in caliber reduction or even occlusion of potentially significant side branches in up to 20% of cases (17). Thus, although exhibiting excellent aneurysm occlusion rates, FD carries significant risk for treatment-related ischemia and should carefully be balanced against SAC and BAC as possibly equivalent treatment option.

Low profile self-expandable (LEO) stents are braided devices composed of a comparatively dense nitinol mesh, exhibiting a smaller pore size than other self-expandable intracranial stents (18). Interestingly, a moderate flow diverting effect of these devices is observable and has already been reported previously (19–21).

In our experience, synergistic treatment of intracranial aneurysms, performing a combination of endosaccular coil occlusion and moderate flow redirection using LEO devices, provides excellent occlusion rates and low therapy-related risk for ischemic complications. Therefore, our retrospective study aimed to explore safety and efficacy of moderately flow-redirecting stent-assisted coiling in incidental and ruptured aneurysms of the intracranial circulation.

## Patients and Methods

This retrospective study was approved by the institutional ethics committee (local IRB nr. AZ 208-15-010062015).

### Patients

We retrospectively reviewed our patient database to identify individuals with aneurysms of the intracranial arterial circulation which have been treated using flow-redirecting stents in combination with coil embolization from March 2015 to February 2017. Patients with incidental, unruptured aneurysms as well as subjects with ruptured aneurysms and subsequent subarachnoid hemorrhage have been included.

Demographic data, localization of the aneurysm, type of stent device, immediate clinical and follow-up angiographic results, as well as complications were recorded.

### Interventional Procedure

Written informed consent was obtained from all patients scheduled for elective endovascular treatment. Patients with acute intracranial hemorrhage gave written informed consent only in case of good clinical condition. Severely affected patients with acute aneurysm rupture were treated justified as emergency indication without informed consent.

Elective patients received pretreatment double antiplatelet loading (ASS 500 mg and clopidogrel 300 mg) followed by standard antiplatelet regimen (ASS 100 mg once per day infinite and clopidogrel 75 mg once per day for 6 months). Clopidogrel resistance was evaluated prior elective intervention. Whenever resistance was present, ticagrelor was administered instead of clopidogrel (90 mg twice per day). In case of acute ruptured aneurysms, ASS premedication (500 mg) was administered during the procedure followed by the double antiplatelet regimen as previously mentioned in elective patients.

All endovascular procedures were performed under general anesthesia using a biplanar angiography (Axiom Artis, Siemens, Germany). Endovascular access was established *via* the right groin using either a 6- or 8-French introducer sheath. In elective procedures without intracranial bleeding, a bolus of heparin (5,000 IE) was administered *via* the sheath. As a flow-redirecting stent, a LEO device (Balt Extrusion, France) was inserted *via* microcatheter access. The selection of stent size was at the discretion of the applying neuroradiologist. Coil embolization of the aneurysms was performed using detachable coil systems in all cases (manufacturers were either Microvention, Stryker, or Codman upon the interventionalists’ discretion). Immediate treatment success was recorded by biplanar angiographic control series.

### Follow-up

Patients with bleeding aneurysms were surveilled at the intensive care unit dependent on their clinical course. Furthermore, all elective patients were monitored on an intensive care unit for at least one night after the intervention. Initial angiographic follow-up was scheduled at 6 months. The extent of occlusion completeness was rated according to the Raymond scale (I—complete occlusion, II—neck remnant, III—residual sac filling, and IV—complete isomorphic persistence of the aneurysm as compared with pre-interventional status).

## Results

### Patients

39 patients (25 females, 14 males) with a total of 40 aneurysms were included in our retrospective evaluation. The average patient age was 54.3 years (range 34–79 years). 32 patients were scheduled for elective treatment, 7 patients had ruptured aneurysms with subsequent subarachnoid hemorrhage. 9 patients were already pretreated with aneurysm coiling and were scheduled of elective re-intervention of dome or neck remnants. Vessel localizations of the aneurysms are listed in Table 1. Distribution of aneurysms was approximately adequate for both sides (17 right, 23 left).

**Table 1:** Localization of treated aneurysms.

Vessel	*n*
Total	40
Anterior communicating artery	13
Middle cerebral artery	12
Internal carotid artery	5
Anterior cerebral artery	3
Basilar artery	2
Vertebral artery	1
Posterior cerebral artery	1
Posterior communicating artery	1
Posterior inferior cerebellar artery	1
Primitive trigeminal artery	1

### Implanted Devices and Technical Feasibility

**Figure 1:**
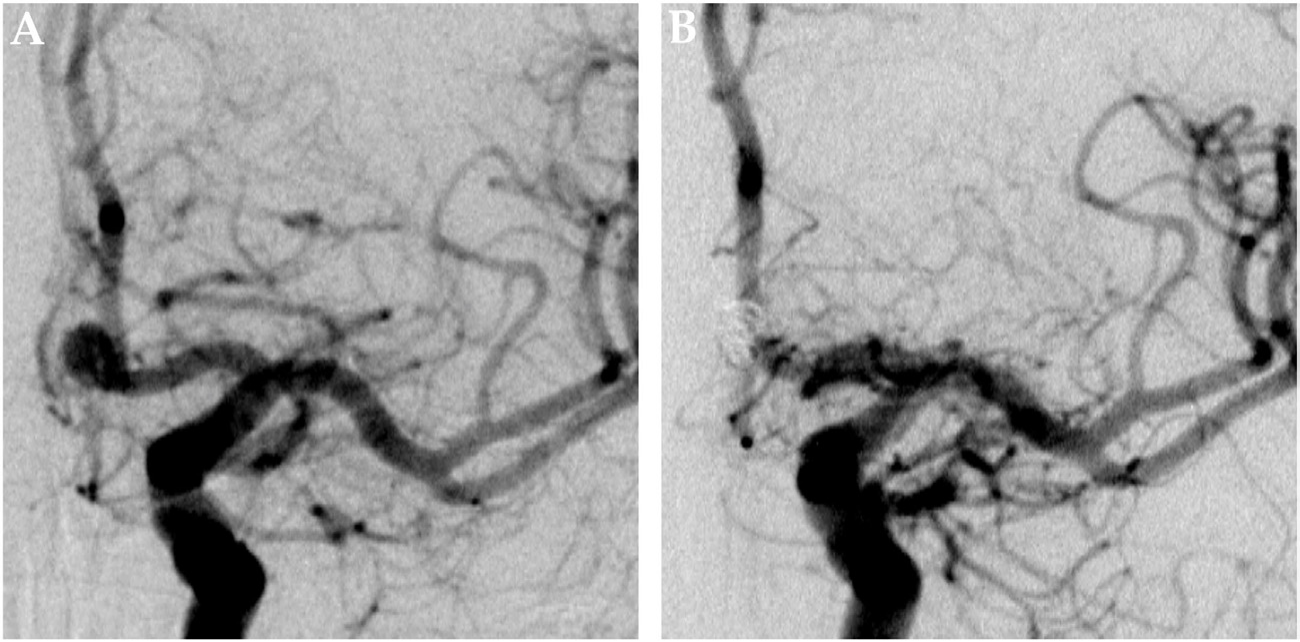
Saccular aneurysm of the left anterior cerebral artery. Panel **(A)** shows a pretreatment DSA image of a saccular aneurysm at the A1–A2 junction of the left ACA in posterior–anterior projection. Panel **(B)** shows the corresponding 6 months posttreatment control DSA image, the aneurysm is completely excluded from the intracranial circulation; the formerly aneurysm-carrying vessel displays regular endoluminal contrast filling and now reveals signs of periprocedural damage.

**Figure 2:**
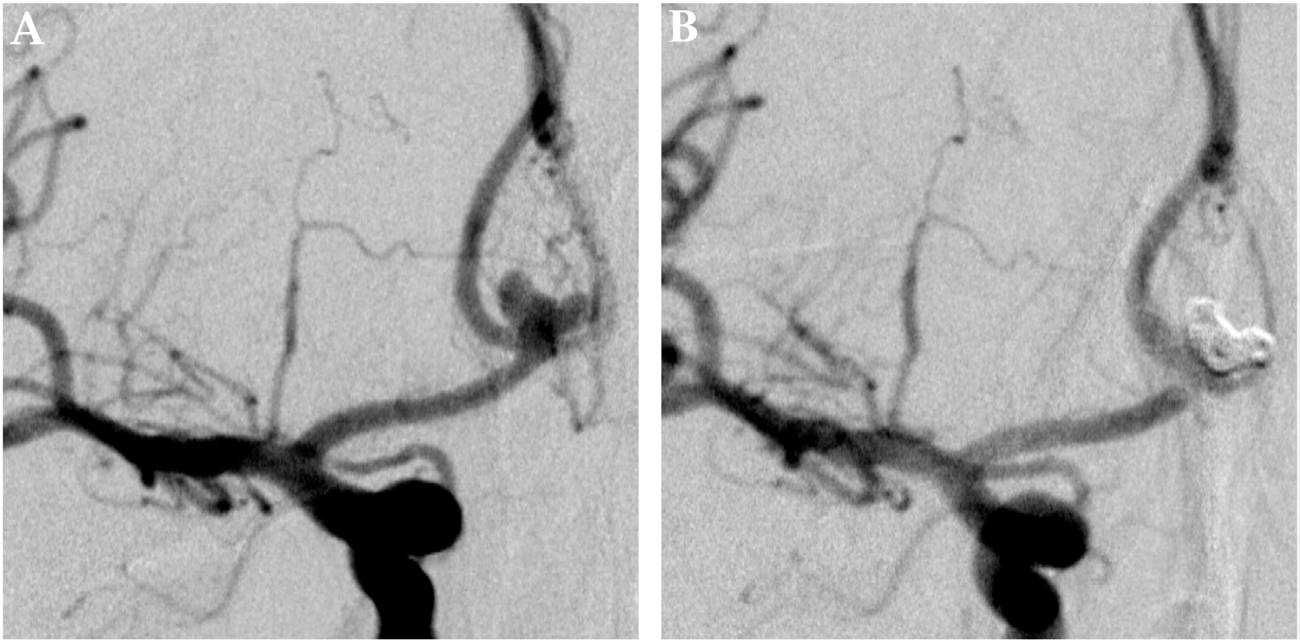
Multilobulated aneurysm of the anterior communicating artery. Panel **(A)** shows a pretreatment DSA image of a lobulated aneurysm of the AcomA in posterior–anterior projection. Panel **(B)** shows the 6 months posttreatment control DSA image, the aneurysm is completely excluded from the intracranial circulation; the formerly aneurysm-carrying vessel displays regular endoluminal contrast filling and now reveals signs of periprocedural damage.

**Figure 3:**
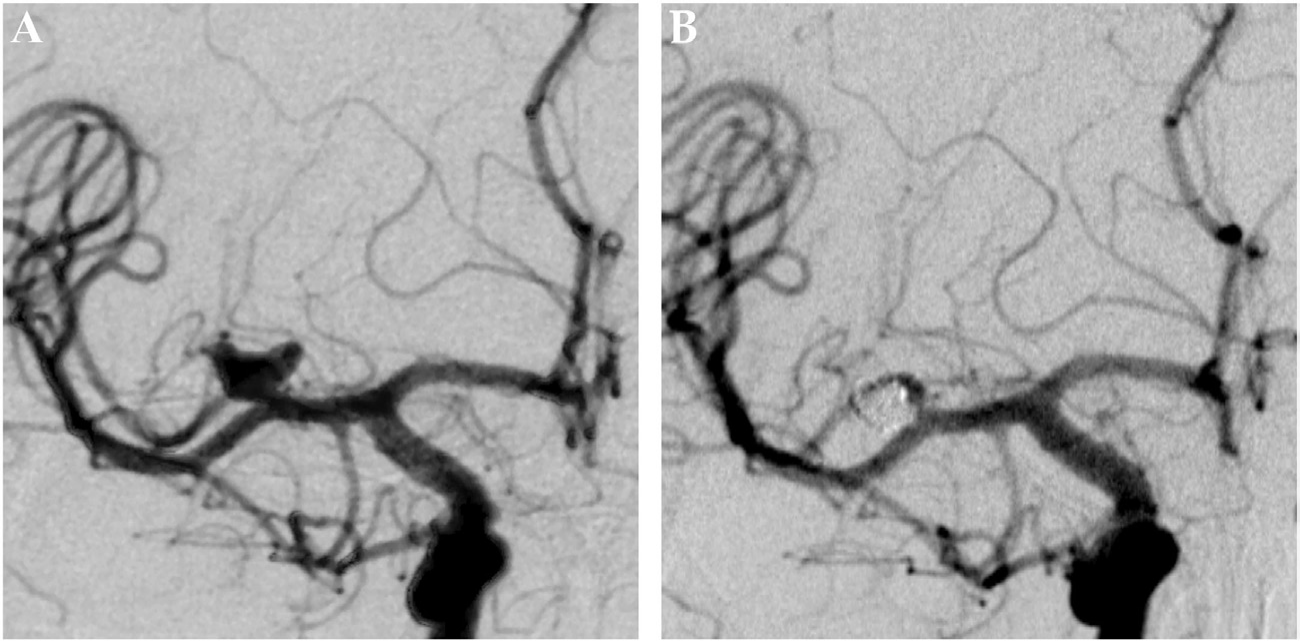
Multilobulated aneurysm of the first branch of the right middle cerebral artery. Panel **(A)** shows a pretreatment DSA image of a multilobulated aneurysm of the first MCA branch on the right side in posterior–anterior projection. Panel **(B)** shows the 6 months posttreatment control DSA image, the aneurysm is completely excluded from the intracranial circulation; the formerly aneurysm-carrying vessel displays regular endoluminal contrast filling and now reveals signs of periprocedural damage.

**Figure 4:**
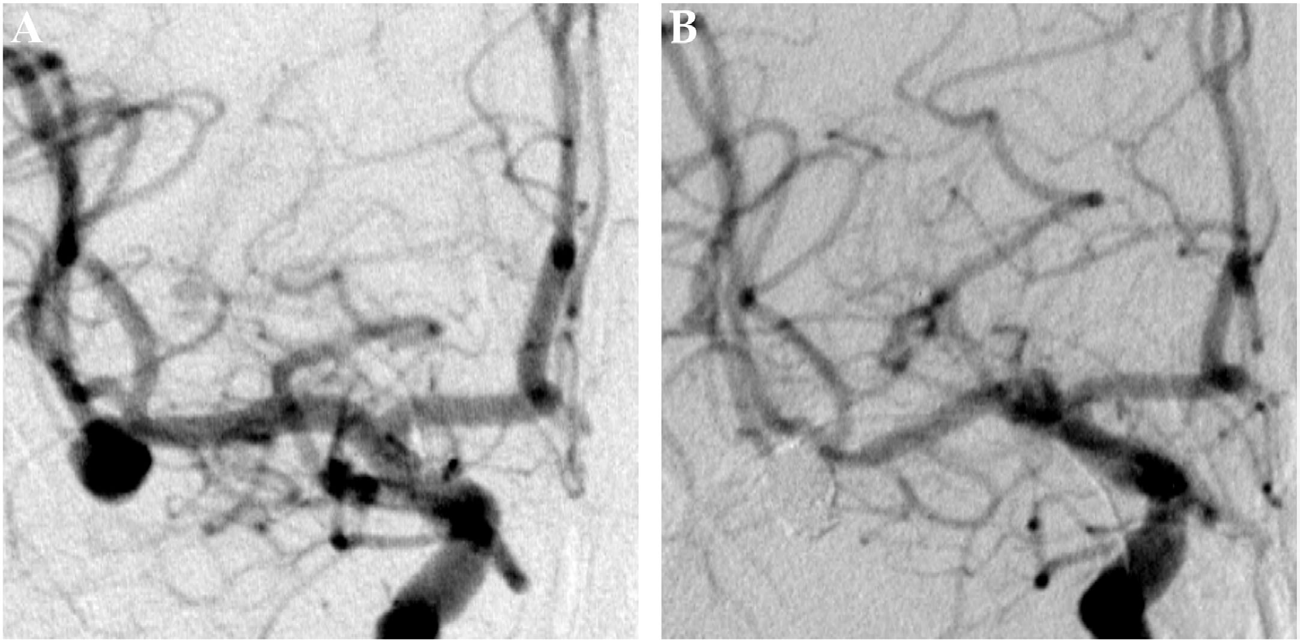
Saccular aneurysm of the right middle cerebral artery trifurcation. Panel **(A)** shows a pretreatment DSA image of a saccular aneurysm of the right-sided MCA-trifurcation in posterior-anterior projection. Panel **(B)** shows the 6months posttreatment control DSA image, nicely demonstrating a complete exclusion of the aneurysm from the intracranial circulation. The formerly aneurysm-carrying vessel displays regular endoluminal contrast filling and reveals now signs of periprocedural damage.

**Figure 5:**
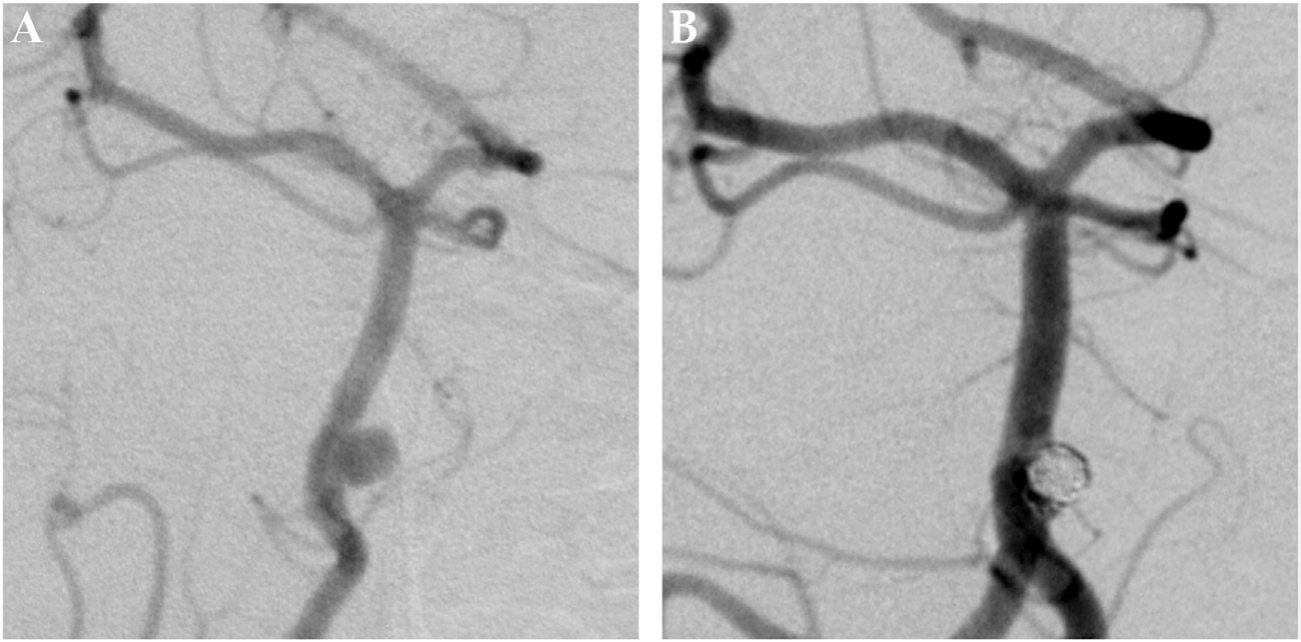
Saccular aneurysm of the basilar artery. Panel **(A)** shows a pretreatment DSA image of a saccular aneurysm of the proximal basilar artery in left anterior oblique projection. Panel **(B)** shows the 6 months posttreatment control DSA image. The formerly aneurysm-carrying vessel displays regular endoluminal contrast filling and now reveals signs of periprocedural damage. The aneurysm is completely excluded from the circulation.

**Figure 6:**
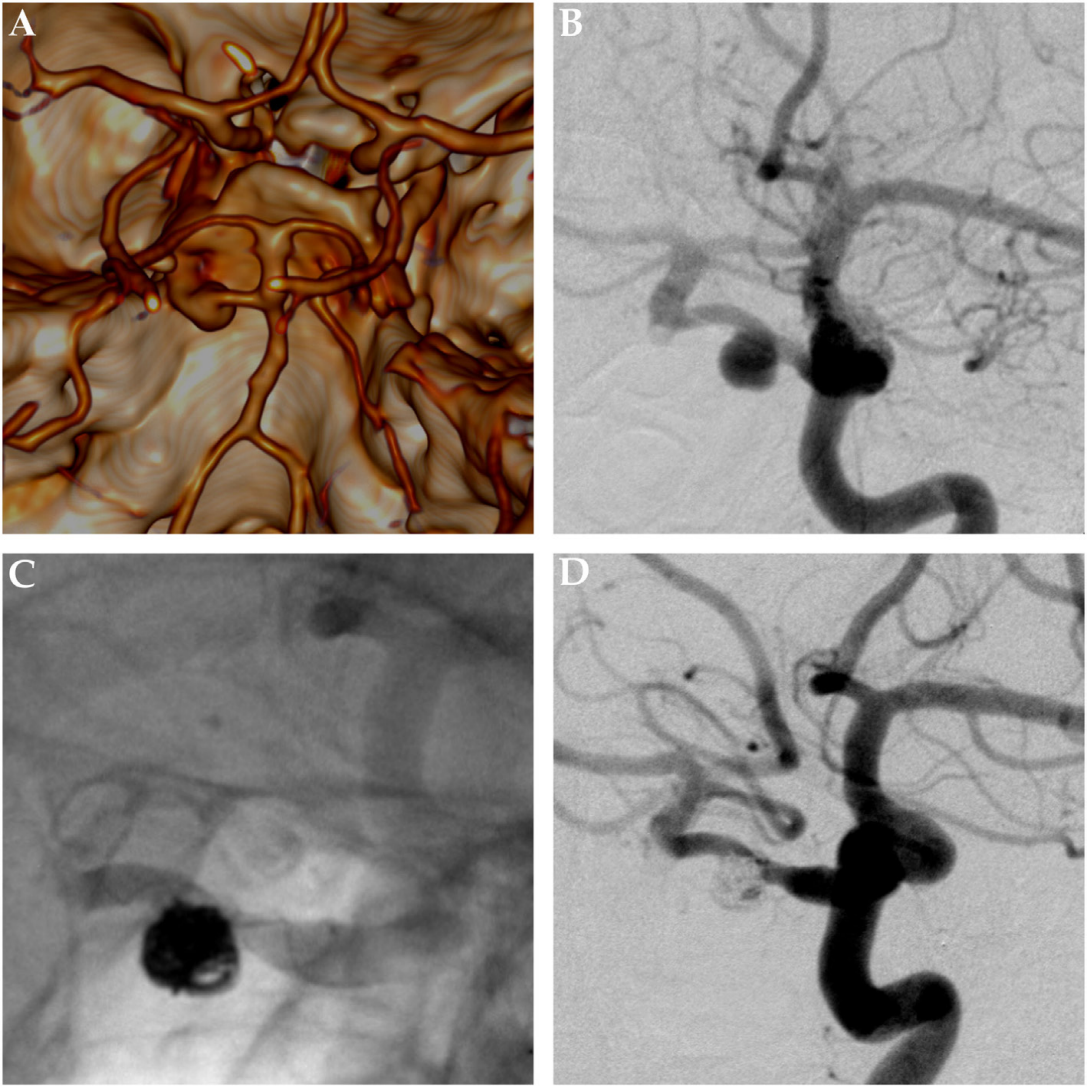
Saccular aneurysm of a primitive trigeminal artery. Panels **(A,B)** show pretreatment images of a saccular aneurysm of a right-sided primitive trigeminal artery aneurysm. Panel **(A)** is the reconstructed and volume rendered CT-angiography. Panel **(B)** gives a posterior–anterior DSA projection. Panel **(C)** shows the nonsubtracted posttreatment control DSA image, nicely demonstrating the helical radiopaque markers of the low profile self-expandable stent and the radiopaque mass of platin microcoils, sufficiently excluding the aneurysm from the intracranial circulation. Panel **(D)** shows the corresponding subtracted DSA image. The formerly aneurysm-carrying vessel displays regular endoluminal contrast filling and now reveals signs of periprocedural damage.

A list of implanted stent devices can be seen in Table 2. The technical application of stents and coils was unproblematic in all cases. No technical defects occurred. Examples for LEO-assisted coil-occusions are provided in Figures 1–6.

**Table 2:** List of implanted flow-redirecting stent devices.

Device	*n*
Total	41
LEO + Baby 2,0/12 mm	9
LEO + Baby 2,0/18 mm	12
LEO + Baby 2,0/25 mm	4
LEO + Baby 2,5/12 mm	2
LEO + Baby 2,5/18 mm	6
LEO + Baby 2,5/25 mm	3
LEO + 3,5/18 mm	4
LEO + 3,5/25 mm	1

*LEO, low profile self-expandable*.

### Complications

Procedure-related complications were observed in 6/39 patients. Acute in-stent thrombosis occurred in two patients in the emergency situation and resolved immediately after local administration of abciximab. Under the assumption that both cases were related to clopidogrel non-response, dual platelet function inhibition regimen was changed to ASA plus ticagrelor (Brilique). Under this regimen, no further thromboembolic complications occurred. One patient scheduled for elective treatment had an iatrogenic intraprocedural coil perforation of the aneurysm with subsequent subarachnoid hemorrhage. In one case of a dissecting vertebral artery aneurysm, acute re-bleeding occurred few days after the initial intervention and was treated successfully by inserting an additional flow diverter. There was one intraprocedural carotid dissection without hemodynamic relevance and no need for specific intervention. Only one groin complication occurred: a femoral arteriovenous fistula resulting from *via*-falsa-puncture had to be treated surgically.

### Follow-up

Angiographic follow-up at 6 months was available in 26/39 patients (27 aneurysms). Complete occlusion (Raymond I) was achieved in 24/27 aneurysms. Small neck remnants (Raymond II) were evident in 3/27 aneurysms. There were no cases with sac remnant or complete persistence of aneurysmal filling (Raymond III and IV). One patient presented with an asymptomatic late onset in-stent occlusion.

### Predictors of Successful Treatment

As a consequence of the comparatively small sample size of our preliminary study, especially with reference to the heterogeneity of the included clinical scenarios, predictors for successful treatment can only be based on empirical values in this context. So far, the presented technique using braided LEO stents in combination with coil occlusion has achieved good short-term outcomes when applied in medium-sized or small-sized intracranial branches of the anterior circulation, beginning with the A1 and M1 segments. Owed to the moderate density of the LEO-Nitinol mesh, aneurysms of larger caliber vessels, for example, the internal carotid artery or the vertebral artery, may not be suitable for this approach.

In the following, we present predictors of successful treatment, based on our experiences using the LEO-coil approach in medium-sized and small-sized intracranial vessels such as the A1/A2 segments and the M1/M2 segments.

In case of initial treatments, complete filling of the aneurysm sac with coils without apparent residual perfusion of the aneurysm dome, exclusive of unsignificant neck remnants, in combination with full coverage of the aneurysm neck by the respective LEO stent, were strongly predictive for persisting occlusion of the aneurysm.

In case of subsequent treatments of aneurysms previously treated with solitary coil occlusion, significant down staging (Raymond IV/III to Raymond II/I) was predictable, if coverage of the aneurysm neck was complete and wall approximation of the respective LEO stent was anatomical.

## Discussion

Intracranial, especially subarachnoid hemorrhage is a serious complication of aneurysms located at the Circle of Willis. To prevent initial bleeding in incidental aneurysms or re-bleeding in ruptured aneurysms, several techniques for the treatment of intracranial aneurysms have been used with varying success rates in the past.

Nowadays, endovascular treatment is favored whenever feasible in the patients specific circumstance (22). Stents in combination with coils are frequently used to prevent herniation of coils into the parent vessel. Several studies using a variety of devices have demonstrated favorable outcomes. For example Gory et al. found rates of 60% complete occlusion and 29.1% neck remnants 6 months after stent-assisted treatment using the Solitaire AB stent (23), albeit the architecture of the Solitaire AB does not allow for redirection of blood flow within the aneurysm-carrying vessel (24).

Stents with flow-redirecting properties are able to further enhance aneurysmal thrombosis by diverting blood flow toward the parent vessel and away from the aneurysmal base (9, 25). Flow diverters in the narrower sense thereby allow aneurysm embolization without placing coils (15). On the other hand, due to their dense woven architecture, they require a stricter antiplatelet regimen to avoid ischemic complications and unintended occlusion of covered side branches is a notorious problem (17). Compared with the non-flow-redirecting Solitaire stent, the SILK device (Balt Extrusion, France)—a flow diverting stent—has been shown to have higher rates of full aneurysmal occlusion with 81.8% on 1-year follow-up (26). Another frequently used flow diverter is the pipeline embolization device. As one example, this device reached full aneurysm occlusion of ~70% in a series of aneurysms of the anterior circulation—although the treated aneurysms were indicated as complex cases (27).

In our opinion, coiling in combination with moderately flow-redirecting stent devices represents a worthwhile middle course between pure coiling and the application of flow diverters in the endovascular treatment of intracranial aneurysms. Hereby, increased risk for recurrence, as observed in sole coil occlusion, is avoided, and ischemic complications become less probable compared with treatment with highly efficient flow diverting devices such as the PED and p64. In comparison with the previously mentioned studies, our own results using LEO stent-assisted coiling impressively demonstrate their superiority in long-term occlusion of aneurysms in combination with coiling. On 6-month follow-up, nearly 90% of our patients showed complete aneurysmal occlusion by means of flow-redirecting stent-assisted coiling. We found only three patients (roughly 10%) to have unsignificant neck remnants, of which one was a pretreated aneurysm which sac reperfusion which could have been “down-graded” to Raymond II stage by re-intervention using LEO stent-assisted recoiling. To this end, we believe that this approach is outstanding in long-term prevention of subarachnoid (re)-bleeding.

Our study has some inherent limitations. First, this is a retrospective approach so that documentation of the data is not standardized and may vary case dependent. Second, our patient collective was quite inhomogeneous as elective patients were included as well as individuals with acute intracranial hemorrhage. To this end, especially data on complications and outcome have to be seen with cautiousness as they may inherently vary between these two groups. Third, there might be a bias in the follow-up data as not all patients presented for 6-month follow-up. We still included those without half year follow-up, as we aimed to assess not only occlusion rates but also technical feasibility of the procedures. In general, 6 months are a relatively short follow-up period and are only partly a predictor for long-term outcome. Fourth, different coil systems were used upon the discretion of the performing interventional radiologist. Nevertheless, we would not expect significant differences in occlusion rates between the different coil systems in our patient collective.

## Conclusion

Our study demonstrates interventional treatment of intracranial aneurysms using braided LEO stents with moderately flow diverting features in combination with intra-aneurysmal coiling to be a technically feasible and promising approach. In our experience, medium-sized and small-sized intracranial arteries, exemplarily the M1/M2 segments and the A1/A2 segments, are suitable target vessels. However, further studies validating our results and providing a comparative analysis between the presented method, solitary coil occlusion, stent-assisted coiling, FD, and flow diverter-assisted coil occlusion are necessary to better define the value of this endovascular technique for clinical practice.

## Ethics Statement

This study was carried out in accordance with the recommendations of the local IRB with written informed consent from all subjects. All subjects gave written informed consent in accordance with the Declaration of Helsinki. The protocol was approved by the Ethikkommission der Medizinischen Fakultät (AZ 208-15-010062015).

## Author Contributions

PV, SS, and UQ performed the analysis and wrote the paper. UQ, UN, MK, DW, and DL performed the interventions and were responsible for patients care and management. RJ was responsible for data management and technical assistance during the procedures. K-TH participated in writing the paper and drafting the concept.

## Conflict of Interest Statement

The authors declare that the research was conducted in the absence of any commercial or financial relationships that could be construed as a potential conflict of interest.
